# Drivers of and Barriers to Behavioural Change to Support Public Health and Social Wellbeing in Mbire District, Zimbabwe

**DOI:** 10.3390/ijerph22091419

**Published:** 2025-09-11

**Authors:** Davison Munodawafa, Pepukai Manjeru, Lioyd Goronga

**Affiliations:** 1 Department of Community Medicine, Midlands State University, Gweru P. Bag 9055, Zimbabwe; 2 Department of Crop Sciences, Midlands State University, Gweru P. Bag 9055, Zimbabwe; 3 Department of Statistics and Operations Research, National University of Science and Technology, Ascot, Bulawayo P.O. Box AC 939, Zimbabwe; lloydgoronga@live.com

**Keywords:** birth registration, exclusive breastfeeding, early childhood development education, open defecation, energy efficiency, social norms, behavioural science, Zimbabwe

## Abstract

Foundational behaviours across health, education, sanitation, and energy use remain suboptimal in Mbire District, Zimbabwe. This qualitative formative study examined drivers of and barriers to five priority behaviours: birth notification and registration (BNR), exclusive breastfeeding (EBF), early childhood development education (ECDE), open-defecation-free (ODF) practices, and efficient use of energy (EUE). Between 15 January and 30 March 2023, we conducted 15 focus group discussions (*n* = 180 participants) and 20 key informant interviews (*n* = 20 participants). Data were thematically analysed in QDA Miner 6 (*Cohen’s κ =* 0.82). Drivers of positive behaviours included leadership support, peer networks, and radio/village meetings, while barriers included bureaucratic requirements, cultural norms, and financial constraints. We recommend a multi-sectoral Social and Behavioural Change (SBC) approach integrating community events, subsidies, and culturally sensitive communication. These findings provide actionable evidence to inform district-level programming and contribute to achieving Zimbabwe’s national development targets and relevant Sustainable Development Goals.

## 1. Introduction

In Zimbabwe, national policies and strategic frameworks have long aimed to support foundational behaviours to support public health and social wellbeing, yet significant implementation gaps persist at the district level. Mbire District, a predominantly rural area in Mashonaland Central Province with a youthful population of approximately 83,720 (63.8% under 25) [1], exemplifies these challenges. Recent data indicate that only 60% of births are registered by age one [2], rates of exclusive breastfeeding (EBF) have fallen to 42% [3], less than half of eligible children are enrolled in Early Childhood Development Education (ECDE) programs [4], 23% of households still practice open defecation [5], and rural access to electricity remains low at 31.6% [6]. These indicators not only fall short of national targets but also undermine progress towards the Sustainable Development Goals (SDGs).

While sector-specific studies have been conducted in Zimbabwe, there is limited evidence based on integrated, multi-domain behavioural analysis at the district level. This study addresses that gap by applying a unified Social and Behavioural Change (SBC) framework to five foundational behaviours, offering a novel, cross-sectoral perspective.

Birth registration is a fundamental right, yet in sub-Saharan Africa, barriers such as high fees, inadequate services, and low awareness impede progress [7,8]. Unregistered children in Zimbabwe face exclusion from education and healthcare, with pronounced disparities by wealth and geography [9]. Similarly, while global EBF rates have improved, Zimbabwe has seen a decline, influenced by cultural practices and socio-economic factors that challenge the message of offering “breast milk only” for the first six months [3,10]. In education, parental and community involvement are critical for the success of ECDE, but involvement is often hampered by low engagement, socio-economic barriers, and structural issues within the education system [4,11].

In the domain of sanitation, open defecation remains a pressing public health issue, contributing to the spread of diarrhoeal diseases and increasing vulnerability to gender-based violence [5,12]. Despite the implementation of Community-Led Total Sanitation (CLTS) initiatives, inadequate infrastructure and funding challenge the goal of achieving Open Defecation-Free (ODF) communities [13]. Finally, in the energy sector, while 62% of Zimbabwean households have electricity, a stark urban–rural divide persists, with most rural communities dependent on biofuels [6]. The energy-consumption patterns of Zimbabwe’s large youth population represent an under-researched area with significant potential for promoting energy efficiency [14].

Past formative studies in Zimbabwe have typically examined drivers of and barriers to positive behaviours within individual domains. However, the interdependence of these behaviours necessitates a more integrated approach; for instance, progress in water, sanitation, and hygiene (WASH) can reinforce health and education outcomes. Our study utilises a qualitative formative approach to generate holistic, actionable insights for a unified Social and Behavioural Change (SBC) strategy in Mbire. The research addresses two primary questions: (1) What are the specific local factors that drive or inhibit each of the five foundational behaviours? and (2) What cross-cutting themes can inform the design of a more coherent, resource-efficient, and impactful multi-sectoral SBC program?

To provide a cohesive analytical lens for these diverse domains, this study is anchored in the Social and Behavioural Change (SBC) framework, which posits that behaviours are shaped by intersecting individual, social, and structural factors. Specifically, we draw on concepts from the Social Ecological Model to explore determinants at multiple levels (individual, family, community, and system) and the COM-B model (Capability, Opportunity, Motivation–Behaviour) to understand how these determinants translate into action. This theoretical approach justifies the selection of these five behaviours, as they share common underlying drivers such as financial barriers (Opportunity), cultural norms (Opportunity), and knowledge gaps (Capability) that can be addressed more efficiently through a unified, multi-sectoral strategy than through siloed programs.

## 2. Materials and Methods

### 2.1. Study Design and Setting

A qualitative formative study was conducted between January and March 2025 in Mbire District, Mashonaland Central Province, Zimbabwe. The district is characterized by a semi-arid climate and dispersed rural settlements, which present logistical challenges for data collection.

### 2.2. Participant Selection

A total of 200 individuals participated in this study, including 180 in 15 FGDs and 20 in 20 KIIs. A multi-stage purposive sampling strategy was employed to ensure representation across key demographics. This non-probabilistic approach was chosen because it is well-suited for an in-depth, exploratory qualitative study aimed at generating rich insights and diagnostic data rather than population-level generalizations. The sample size was determined by the principle of thematic saturation, which was deemed to have been reached during analysis.

First, wards were stratified as rural or peri-urban. Within these strata, participants were recruited to meet quotas for sex, age group (18–24, 25–34, 35+), educational attainment, and occupation to ensure that a diverse range of perspectives was included.

### 2.3. Data Collection and Analysis

#### 2.3.1. Data Collection Methods

A team of trained enumerators collected data through focus group discussions (FGDs) and key informant interviews (KIIs) using KoBoToolbox version 2.023.04b on tablets to capture and record responses in real time. Audio recordings were transcribed verbatim, and those conducted in Shona were translated into English, ensuring high accuracy and fidelity to participant narratives.

#### 2.3.2. Data Analysis

The translated transcripts from the FGDs and KIIs were imported into the qualitative data analysis software QDA Miner 6 for thematic analysis. A hybrid coding approach was employed. Initially, a deductive codebook was developed based on themes from the literature review. This was subsequently expanded with inductive codes that emerged directly from the participant narratives. This process resulted in a comprehensive codebook delineating specific behavioural drivers (e.g., leadership advocacy, radio communication, community support) and barriers (e.g., documentation complexity, cultural norms, financial constraints).

To ensure analytical rigour, two independent researchers coded 10% of the transcripts. The resulting codes were compared, and any discrepancies were discussed and resolved to refine the codebook definitions. Inter-rater reliability was then calculated using Cohen’s kappa, which yielded a score of 0.82, indicating a high level of agreement between the coders according to Landis and Koch’s benchmark scale. Following this, the primary researcher coded the remaining transcripts. The analysis involved quantifying code frequency (the number of times a code was applied) and case coverage (the percentage of FGDs/KIIs in which a code appeared) to systematically identify and compare the most salient themes across the five behavioural domains.

#### 2.3.3. Explanation of “Case Coverage”

The term ‘case coverage’ in the results refers to the percentage of qualitative data-collection sessions (total of 15 FGDs and 20 KIIs, *n* = 35) in which a specific theme or code was identified. For instance, a case coverage of 85.7% indicates that a particular driver or barrier was mentioned in 30 out of the 35 total qualitative sessions. The recurring percentages (e.g., 71.4%, 42.9%) correspond directly to the frequency of themes across these 35 sessions, providing a semi-quantitative measure of thematic prevalence.

### 2.4. Ethical Considerations

The study protocol was approved by the Institutional Review Board of Midlands State University (Approval No. MSU/IRB/2023/112, approved 15 December 2022). Informed consent was obtained from all adult participants before their involvement in the study, and this was documented on the questionnaire. The study ensured the anonymity and confidentiality of all participants. No individuals under the age of 18 were included. Anonymized datasets and codebooks are available from the corresponding author upon reasonable request.

Potential biases, such as social desirability bias in self-reported practices, were mitigated by ensuring anonymity and using trained, neutral facilitators who fostered an open and non-judgmental environment. To minimize recall bias, questions focused on recent or current practices. The full interview guides are available in Appendix B and Appendix C.

## 3. Results

### 3.1. Participant Demographics

Of the 200 participants (180 from FGDs and 20 from KIIs), 102 (51.0%) were female and 98 (49.0%) were male. The ages of participants ranged from 18 to 65 years, with a median age of 29 (IQR 22–37). In terms of education, 37% had completed primary education or less, 52% had completed secondary education, and 11% had attained tertiary education. The primary occupations included farming (45%), informal trade (20%), health/village health work (10%), education (8%), and leadership roles (7%). A detailed breakdown of participant demographics is provided in Appendix A, Table A1.

### 3.2. Birth Notification and Registration (BNR)

Qualitative analysis revealed that traditional leaders were a primary driver of BNR, with 85.7% of FGDs/KIIs crediting them with championing registration drives. As one participant noted, *“The headmen sometimes go spreading the word that parents should take their children’s birth certificates, and we also get the information at the clinic.”* Clinic-based health talks and local radio announcements also reinforced the importance of BNR for 42.9% of the groups. Qualitative analysis suggested that education level was a key determinant of BNR uptake. The deployment of mobile civil-registration units was another key facilitator, although their reach was limited to 14.3% of wards.

The most significant barrier, cited in 85.7% of discussions, was the complexity of documentation. A participant explained, *“They need both parents and their ID numbers,”* a requirement that, coupled with transport costs, proved prohibitive for many. A KII participant from the civil registry office noted, *“Cases vary, and we can have a situation where one parent is deceased… a death certificate... is required... if both of them are deceased both the death certificates are required and two near relatives are needed to be witnesses.”* This highlights the bureaucratic challenges families face.

This was particularly challenging when initial birth records were missing. Distance was another obstacle for 28.6% of groups, with one respondent sharing, *“sometimes during the rainy season the rivers can be full and we have to wait till we are able to cross.”* Furthermore, 85.7% of groups reported that cultural preferences for home births, which often lack formal notification, hindered the registration process. Table 1 summarises the drivers of and barriers to birth notification and registration.

### 3.3. Exclusive Breastfeeding (EBF)

Community radio campaigns were a major driver of EBF, being mentioned in 85.7% of FGDs/KIIs. Counselling by health workers at postnatal clinics provided essential practical guidance for 42.9% of groups. One mother shared, *“Health workers educate mothers on how to do things healthily regarding breastfeeding.”*

However, the cultural practice of early complementary feeding was a dominant barrier, mentioned in 85.7% of discussions. A participant explained, *“Babies are given porridge and maheu [a traditional beverage],”* often by older female relatives. A perceived insufficient milk supply, a concern for 71.4% of groups, was often linked to maternal nutrition challenges. Additionally, 28.6% of participants noted resistance from family members, particularly grandmothers, who distrusted the *“breast milk only” message. One mother in an FGD elaborated on this family pressure: “It becomes difficult because some mothers may have breasts that will not be producing milk at all that’s why they decide to give a baby other foods.”* Another added, *“Mother-in-laws are number one in not supporting EBF, in a week they give water and say the child is thirsty.”* Table 2 summarises drivers of and barriers to EBF.

### 3.4. Early Childhood Development Education (ECDE)

Community mobilization through School Development Committees (SDCs) was a key driver, with 71.4% of discussions highlighting their role in advocating for ECDE enrolment. Parental engagement in home-based learning activities was seen as pivotal for school readiness by 85.7% of groups. Financial support from NGOs or community fundraising enabled 35.0% of families to enrol their children.

The most pervasive barrier, emphasized by 85.7% of participants, was financial constraints. As one parent stated, the primary issue is a *“Lack of money to pay school fees.”* This financial barrier was a constant refrain. A parent in an FGD stated simply, *“Parents will not be having money, the child will not be putting the pressure to go to school.”* This led to delayed enrolment or irregular attendance. A lack of awareness regarding the importance of ECDE was noted by 28.6% of groups, with a common belief that formal schooling should only begin at age seven. Cultural narratives suggesting that *“children need to help with chores before learning”* also emerged as a barrier in 28.6% of FGDs. Table 3 summarises drivers of and barriers to ECDE.

### 3.5. Open-Defecation-Free (ODF) Practices

Community-Led Total Sanitation (CLTS) workshops were a powerful driver, galvanizing 78.6% of wards to adopt ODF practices. Public commitments to ODF targets by traditional leaders, noted in 64.3% of groups, further reinforced these efforts. School-based sanitation clubs also played a role in instilling hygienic habits in children, as mentioned by 50.0% of groups.

Despite these initiatives, a shortage of construction materials for latrines was a major constraint for 71.4% of participants. Seasonal water scarcity was a challenge for 57.1% of wards. Furthermore, 42.9% of groups described cultural taboos against sharing latrines as a significant barrier. One respondent explained, *“Some people do not want to go to the toilet... more often in forests, in their lands.”* A village leader explained the challenge: *“Some people do not have a toilet and some are lazy to build toilets and some do it because it will be their behavior.”* Another participant highlighted the lack of resources: *“They lack cement so people procrastinate to have toilets.”* Table 4 summarises drivers of and barriers to ODF.

### 3.6. Efficient Use of Energy (EUE)

Awareness campaigns on the benefits of clean cookstoves reached 71.4% of communities. Government and NGO subsidies were a key driver for 50.0% of participants. The local availability of fuel-efficient stoves and solar lanterns in 42.9% of wards also facilitated adoption.

Nevertheless, the high residual cost of even subsidized stoves remained a significant barrier for 78.6% of the poorest households. A participant noted, *“Trying to buy gas stoves is very hard for us.”* Cultural preferences for traditional three-stone fires prevailed in 57.1% of discussions. Additionally, 64.3% of participants reported a lack of local maintenance services, with one explaining, *“The issue of tsotso stoves was taught... but we don’t have enough knowledge because they did not train us.”*

An environmental officer explained the community’s reliance on traditional methods: *“The wide use is firewood… the reason is it is readily available… the entire district… is covered by forest.”* However, a participant in an FGD highlighted the trade-off: *“Firewood is fetched far away, it now needs a cart. If you don’t have, you don’t get firewood.”* Table 5 summarises drivers of and barriers to EUE.

## 4. Discussion

This integrated analysis across five distinct yet interconnected domains in Mbire District reveals several cross-cutting themes that are critical for the development of an effective multi-sectoral SBC strategy.

### 4.1. Cross-Cutting Enablers

Community leadership consistently emerged as the most influential driver across all five behaviours. The active involvement of traditional leaders in mobilizing communities, endorsing targets, and legitimizing new practices was pivotal. This finding aligns with research highlighting the crucial role of community leaders in advocating for civil registration and other public health initiatives in rural African contexts [15].

Targeted communication, particularly through local radio, village meetings, and school clubs, proved highly effective in amplifying messages and sharing peer testimonials. This underscores the importance of using trusted, accessible channels to disseminate information and foster social support for behaviour change.

Financial and material support, whether provided by NGOs, the government, or through community fundraising, was a critical enabler when it was available. This highlights the reality that for many households in Mbire, the intention to adopt positive behaviours is often constrained by economic realities. Comparable integrated SBC interventions in Malawi and Zambia have similarly demonstrated that leveraging community leadership and trusted communication channels can accelerate the adoption of multiple health and social behaviours simultaneously [16].

### 4.2. Persistent Barriers

Financial and logistical constraints, including fees, transport costs, and material costs, were the most pervasive obstacles across all domains. This suggests that SBC strategies must be paired with structural interventions that address the economic determinants of behaviour.

Bureaucratic hurdles, especially complex documentation requirements for birth registration, were a significant deterrent. This indicates a need for streamlining of administrative processes to make essential services more accessible.

Deeply entrenched cultural norms, from traditions of early weaning to latrine-sharing taboos and traditional cooking practices, continue to undermine the adoption of new behaviours. This finding reinforces the need for culturally sensitive approaches that engage with, rather than ignore, local beliefs and practices.

Our findings echo the domain-specific literature from Zimbabwe [2,14,15] but offer a unique, integrated perspective. By examining these five behaviours under a single SBC framework, we highlight the potential for synergistic interventions. For example, synchronizing mobile birth-registration services with immunization campaigns and cookstove-distribution events, all under the patronage of local leaders, could maximize reach and efficiency. Similarly, coupling financial subsidies with capacity-building, such as hands-on latrine-construction demonstrations or cookstove-maintenance training, can enhance the sustainability of these interventions.

### 4.3. Limitations of the Study

Recall bias was minimized by focusing on recent behaviours; however, some under- or over-reporting may still have occurred. Social desirability bias was mitigated through assurances of confidentiality and neutral facilitation but cannot be entirely excluded. Selection bias may have arisen from the purposive sampling of key informants and FGD participants, although efforts were made to include diverse perspectives, as detailed in the methods section.

### 4.4. Future Research Directions

Future research should explore the long-term impact of integrated SBC strategies on behavioural outcomes in Mbire and similar contexts. Longitudinal studies could track changes in social norms and behaviours over time. Further investigation into the cost-effectiveness of multi-sectoral versus single-sector interventions would also provide valuable insights for policymakers and program implementers. Future work should also examine how integrated SBC evidence can be translated into district and national policy frameworks, ensuring that behavioural insights inform both strategic planning and resource allocation.

## 5. Conclusions

This formative qualitative study highlights the complex interplay of socio-cultural, economic, and infrastructural factors influencing five priority health and social behaviours in rural Zimbabwe. By applying an integrated Social and Behaviour Change framework, we identified both cross-cutting enablers—such as engagement of community leadership and NGO support—and persistent barriers, including entrenched cultural norms and resource constraints. These insights underscore the need for multi-sectoral, context-specific interventions that address structural determinants while leveraging existing community assets. Future programmes should adopt integrated SBC approaches that can be adapted across behaviours and settings, ensuring sustainability and scalability. The lessons from this study offer a practical blueprint for policymakers, implementers, and researchers seeking to accelerate progress towards national and global public health targets.

## Figures and Tables

**Table 1:** Drivers of and barriers to birth notification and registration.

Driver	Case Coverage	Barrier	Case Coverage
Leadership advocacy at village level	85.7% (*n* = 30/35)	Complex documentation requirements	85.7% (*n* = 30/35)
Mobile registration units	14.3% (*n* = 5/35)	Long travel distances, impassable roads	28.6% (*n* = 10/35)
Clinic-based radio announcements	42.9% (*n* = 15/35)	Cultural preference for home births	85.7% (*n* = 30/35)

**Table 2:** Drivers of and barriers to exclusive breastfeeding.

Driver	Case Coverage	Barrier	Case Coverage
Community radio campaigns	85.7% (*n* = 30/35)	Early supplementary feeding by relatives	85.7% (*n* = 30/35)
Health-worker counselling at clinics	42.9% (*n* = 15/35)	Perceived low maternal milk supply	71.4% (*n* = 25/35)
Peer support and testimonials	42.9% (*n* = 15/35)	Limited family support and traditional beliefs	28.6% (*n* = 10/35)

**Table 3:** Drivers of and barriers to early childhood development education.

Driver	Case Coverage	Barrier	Case Coverage
Community mobilization via SDC meetings	71.4% (*n* = 25/35)	School fees, uniform costs, and transport costs	85.7% (*n* = 30/35)
Parental engagement in home-based learning	85.7% (*n* = 30/35)	Low perceived importance of ECDE	28.6% (*n* = 10/35)
NGO-subsidized fees and uniforms	35.0% (*n* = 12/35)	Cultural belief in prioritizing chores over school	28.6% (*n* = 10/35)

**Table 4:** Drivers of and barriers to open-defecation-free practices.

Driver	Case Coverage	Barrier	Case Coverage
CLTS trigger workshops	78.6% (*n* = 27/35)	Shortage of construction materials	71.4% (*n* = 25/35)
Leadership endorsement of ODF targets	64.3% (*n* = 22/35)	Seasonal water scarcity	57.1% (*n* = 20/35)
School-based sanitation clubs	50.0% (*n* = 18/35)	Cultural taboos around latrine sharing	42.9% (*n* = 15/35)

**Table 5:** Drivers of and barriers to efficient use of energy.

Driver	Case Coverage	Barrier	Case Coverage
Awareness campaigns on fuel-efficient cookstoves	71.4% (*n* = 25/35)	High residual cost despite subsidies	78.6% (*n* = 27/35)
Financial incentives/subsidies	50.0% (*n* = 18/35)	Cultural preference for traditional cooking	57.1% (*n* = 20/35)
Availability of clean cookstoves and solar kits	42.9% (*n* = 15/35)	Lack of local maintenance and spare parts	64.3% (*n* = 22/35)

## Data Availability

Anonymized datasets and codebooks are available from the corresponding author upon reasonable request.

## Appendix A

**Table A1:** Participant Demographics (*n* = 200).

Characteristic	Category	*n* (%)
Gender	Female	102 (51.0)
	Male	98 (49.0)
Age	Median (IQR)	29 (22–37)
Education	Primary or Below	74 (37.0)
	Secondary	104 (52.0)
	Tertiary	22 (11.0)
Occupation	Farming	90 (45.0)
	Informal trade	40 (20.0)
	Health/Village Health Workers	20 (10.0)
	Education	16 (8.0)
	Leadership	14 (7.0)
	Other	20 (10.0)

## Appendix B

FOCUS GROUP DISCUSSION GUIDE
SECTION A: DEMOGRAPHIC INFORMATION

Participant’s information

Age

Gender

Marital status

Geographical location (rural, urban, farm, mining)

Educational qualification

Occupation

Family size

Family income

Religion

SECTION B: BIRTH NOTIFICATION AND REGISTRATION

1. Barriers and drivers

What is your understanding of birth notification and registration?

Why is birth notification and registration important?

What is the process of birth notification and registration?

What are the factors that facilitate birth notification and registration?

Probe culture, religion, community leaders

What are the factors that make it difficult to notify and register a birth?

Probe culture, religion, community leaders, knowledge

Explain other factors that influence birth notification

Probe migration, process

2. SBC communication platforms

Explain where/how you get information on birth notification and registration information in your community?

Probe radio, television, church, awareness campaigns etc.

What are the most common communication channels preferred (most impact) and why?

How best can organisations disseminate information on BR in your community?

3. Landscape mapping

Please give details of organisations that are working to promote BR in this community and their role

Probe -name, area of operation, activities by the organisation, effectiveness

SECTION C: EXCLUSIVE BREAST FEEDING

1. Barriers and drivers

What is your understanding of EBF?

What do you think are the benefits of EBF?

What would make it easier/difficult for mothers to breastfeed exclusively?

Probe Role of husband, parents, in-laws, health care workers, care groups, work/roles etc.

Why do you think mothers give other foods (porridge, water, traditional medicines etc) besides breast milk before the age of 6 months?

Probe role of culture, tradition, beliefs, baby factors etc.

Please explain the cultural taboos and rules that promote or hinder exclusive breastfeeding

Explain other factors that affect EBF

Probe social media, role models, migration

2. SBC communication platforms

Where/How do you get information on exclusive breast feeding in your community?

Probe radio, television, church, awareness campaigns etc.

What are the most common communication channels preferred (most impact) and why?

How best can organisations disseminate information on EBF in your community?

3. Landscape mapping

Please explain organisations that are working to promote EBF in this community and their role—Probe -name of organisation, area of operation, activities by the organisation, effectiveness

SECTION D: EARLY CHILDHOOD DEVELOPMENT

1. Barriers and drivers

What is ECDE? Probe benefits of ECDE, what is being done well in ECDE

How are parents/guardians involved in ECDE?

Probe role, how they facilitate,

What makes it easier for parents/guardians to be involved in ECDE?

Probe behavioural, social norms, culture, religion, community leaders, family size

What makes it difficult for parents/guardians to be involved in ECDE?

Probe behavioural, social norms, culture, religion, community leaders, family size

What do you think can be done better to improve parental involvement in ECDE?

Probe role of Ministry, School administrator, community

2. SBC communication platforms

Where/How do you get information on parental involvement in ECDE in your community?

Probe radio, television, church, awareness campaigns etc.

What are the most common communication channels (When disseminating information on parental involvement in ECDE) preferred (most impact) and why?

How best can organisations disseminate information on parental involvement in ECDE in your community?

3. Landscape mapping

Please explain organisations that are working to promote parental involvement in ECDE in this community and their role—Probe -name of organisation, area of operation, activities by the organisation, effectiveness

SECTION E: OPEN DEFECTION-FREE COMMUNITIES

1. Barriers and drivers

What is your understanding of OD?

To what extent do you view OD as a problem in your community? Why?

What are the major causes of (what encourages) OD in this community?

Probe—Who practices OD, reasons, knowledge of consequences (short and long term), group influence

Which groups are most influential in enforcing ODF (proper use of toilets) these norms, practices, and behaviours? Explain how

Probe role of traditional leaders, religious leaders, group influence etc.

Do communities in general comply with these social/cultural norms, practices, and behaviours ? YES/NO—Give reasons for your answer.

How does educational and income levels influence attitudes towards toilet use?

What are the solutions to the challenge of OD (adopt toilet use)?/What can help prevent OD (adopt toilet use)

Probe values, social norms, cultural/religious and other practices, personal preference, attitudes regarding hygiene (by fathers, boys, women, PLWD, girls, adolescent girls, the disabled and children), role of legislation

2. SBC communication platforms

Where/How do you get information on proper use of toilets/avoiding OD in your community?

Probe radio, television, church, awareness campaigns etc.

What promotional strategies, activities, messages, and message channels are most likely to increase adoption?

How best can organisations disseminate information on proper use of toilets/avoiding OD in your community?

3. Landscape mapping

Please explain organisations that are working to promote parental involvement in ECDE in this community and their role—Probe -name of organisation, area of operation, activities by the organisation, effectiveness

SECTION F: EFFICIENT USE OF ENERGY

1. Barriers and drivers

In your own understanding, what does the term energy mean to you? (Probe to identify common types and/or sources)

Can you share your thoughts regarding the importance of the following different types of energy: (i) home energy, (ii) local energy and (iii) energy efficiency? (knowledge and perceptions on energy, role in mitigating the effects of climate change through efficient use of energy)

What experiences/issues do you face concerning promoting efficient energy use in your homes? Probe: Knowledge, attitudes, locus of control,

What are some of the positive and negative experiences concerning energy use in your homes? Explain what caused these

What challenges do you face concerning energy use in your homes?

What are your fears concerning energy use in your neighbourhood?

What is the role of the household head(s), children, elders concerning how you use energy in the homes? What have they done to control how you use energy in your homes?

(Would you now consider yourself to have a responsibility on how energy is used in your homes? If not, what would you do to save energy if you had the responsibility?)

Can you share how management energy use in your home is done? Probe

(What motivates you to manage energy at home?)

What strategies are employed in conserving energy in homes or Locality by local authorities?

Do you believe there are benefits of using energy efficient measures? Probe: What are they?

What do you think are the roles of government, agencies, officials, local leadership concerning energy and energy use in your homes and locality?

What laws or regulations do you know concerning energy and energy use in your locality?

In your own opinion, what are some of the implications of global changes (things happening in other parts of the world) on efficient use of energy by children and young people? Probe:

Globalization (interconnectedness and inter dependentness of the world), technological innovations and increasing population, migration, urbanization etc.

Can you share your suggestions and recommendations on how authorities can make it easier for children and young people to use energy efficiently?

Preferred learning methods vs. contemporary methods

Policy and governance

2. SBC communication platforms

Where/How do you get information on Efficient energy use in your community?

Probe:—Radio, TV, newspapers, local leadership, authorities (EMA), community leaders, school teachers, other young people)

What promotional strategies, activities, messages, and message channels are most likely to increase adoption of behaviours related to efficient use of energy?

How best can organisations disseminate information on Efficient use of energy in your community?

3. Landscape mapping

Please explain organisations that are working to promote efficient use of energy in this community and their role—Probe -name of organisation, area of operation, activities by the organisation, effectiveness

From your own experiences, what/who do you consider to be reliable sources of information concerning energy and energy use by children and young people in your locality. Probe: Radio, TV, newspapers, local leadership, authorities (EMA), community leaders, school teachers, other young people)

Can you rate how important each of these information sources are to you (high, moderate, low)

Why are these important to you? (Probe on possible reasons e.g., language, channel, trust, accuracy, feedback, accessibility, relevance; including some suggestions to improve)

## Appendix C

KEY INFORMANT INTERVIEWS (KIIs)
SECTION A: DEMOGRAPHIC INFORMATION

Participant information

Age

Occupation

Number of years in the position

Educational qualifications

Gender

SECTION B: BIRTH REGISTRATION

1. Barriers and drivers

What is your understanding of birth notification and registration?

Why is birth notification and registration important?

What is the process of birth notification and registration?

What are the factors that facilitate birth notification and registration?

Probe culture, religion, community leaders, legislation, migration, globalisation

What are the factors that make it difficult to notify and register a birth?

Probe culture, religion, community leaders, knowledge, legislation, migration, globalisation

Explain other factors that influence birth notification

Probe migration, process

2. SBC communication platforms

Explain where/how you get information on birth notification and registration information in your community?

Probe radio, television, church, awareness campaigns etc.

What are the most common communication channels preferred (most impact) and why?

How best can organisations disseminate information on BR in your community?

3. Landscape mapping

(a) Please give details of organisations that are working to promote BR in this community and their role

4. SBC skills gap analysis (for government workers, NGO/CSO staff)

Please explain the skills that you have concerning Social and behavioural issues around BR. Probe formal and informal training

What are the skills related to Social and behavioural change in BR would make your job easier?

SECTION C: EXCLUSIVE BREASTFEEDING

1. Barriers and drivers

What is your understanding of EBF?

What do you think are the benefits of EBF?

What would make it easier/difficult for mothers to breastfeed exclusively?

Probe Role of husband, parents, in laws, health care workers, care groups, work/roles, policies, role of social media etc.

Why do you think mothers give other foods (porridge, water, traditional medicines etc) besides breast milk before the age of 6 months?

Probe role of culture, tradition, beliefs, baby factors etc, policies,

Please explain the cultural taboos and rules that promote or hinder exclusive breastfeeding

Explain other factors that affect EBF

Probe social media, role models, migration

2. SBC communication platforms

Where/How do you get information on exclusive breast feeding in your community?

Probe radio, television, church, awareness campaigns etc.

What are the most common communication channels preferred (most impact) and why?

How best can organisations disseminate information on EBF in your community?

3. Landscape mapping

Please explain organisations that are working to promote EBF in this community and their role—Probe -name of organisation, area of operation, activities by the organisation, effectiveness

4. SBC skills gap analysis (for government workers, NGO/CSO staff)

Please explain the skills that you have concerning Social and behavioural issues around EBF. Probe formal and informal training

What are the skills related to Social and behavioural change in EBF would make your job easier?

SECTION D: EARLY CHILDHOOD DEVELOPMENT

1. Barriers and drivers

What is ECDE? Probe benefits of ECDE, what is being done well in ECDE

How are parents/guardians involved in ECDE?

Probe role, how they facilitate,

What makes it easier for parents/guardians to be involved in ECDE?

Probe behavioural, social norms, culture, religion, community leaders, policies

What makes it difficult for parents/guardians to be involved in ECDE?

Probe behavioural, social norms, culture, religion, community leaders, policies

What do you think can be done better to improve parental involvement in ECDE?

Probe role of Ministry, School administrator, community

2. SBC communication platforms

Where/How do you get information on parental involvement in ECDE in your community?

Probe radio, television, church, awareness campaigns etc.

What are the most common communication channels (When disseminating information on parental involvement in ECDE) preferred (most impact) and why?

How best can organisations disseminate information on parental involvement in ECDE in your community?

3. Landscape mapping

Please explain organisations that are working to promote parental involvement in ECDE in this community and their role—Probe -name of organisation, area of operation, activities by the organisation, effectiveness

4. SBC skills gap analysis (for government workers, NGO/CSO staff)

Please explain the skills that you have concerning Social and behavioural issues around parental involvement in ECDE. Probe formal and informal training

What Social and behavioural change skills concerning parental involvement in ECDEF would make your job easier/will result in increase in parental involvement in ECDE?

SECTION E: OPEN DEFECATION FREE COMMUNITIES

1. Barriers and drivers

What is your understanding of OD?

To what extent do you view OD as a problem in your community? Why?

What are the major causes of (what encourages) OD in this community?

Probe—Who practices OD, reasons, knowledge of consequences (short and long term), group influence

Which groups are most influential in enforcing ODF (proper use of toilets) these norms, practices, and behaviours? Explain how

Probe role of traditional leaders, religious leaders, group influence etc.

Do communities in general comply with these social/cultural norms, practices, and behaviours ? YES/NO—Give reasons for your answer.

How does educational and income levels influence attitudes towards toilet use?

What are the solutions to the challenge of OD (adopt toilet use)?/What can help prevent OD (adopt toilet use)

Probe values, social norms, cultural/religious and other practices, personal preference, attitudes regarding hygiene (by fathers, boys, women, PLWD, girls, adolescent girls, the disabled and children), role of legislation

2. SBC communication platforms

Where/How do you get information on proper use of toilets/avoiding OD in your community?

Probe radio, television, church, awareness campaigns etc.

What promotional strategies, activities, messages, and message channels are most likely to increase adoption?

How best can organisations disseminate information on proper use of toilets/avoiding OD in your community?

3. Landscape mapping

(a) Please explain organisations that are working to promote parental involvement in ECDE in this community and their role—Probe -name of organisation, area of operation, activities by the organisation, effectiveness

4. SBC skills gap analysis (for government workers, NGO/CSO staff)

Please explain the skills that you have concerning Social and behavioural issues around promoting open defecation free communities/proper and consistent use of latrines. Probe formal and informal training

What Social and behavioural change skills would make your job easier/will result in increase in proper and consistent use of latrines?

SECTION F: EFFICIENT USE OF ENERGY

1. Barriers and drivers

In your own understanding, what does the term energy mean to you? (Probe to identify common types and/or sources)

Can you share your thoughts regarding the importance of the following different types of energy: (i) home energy, (ii) local energy and (iii) energy efficiency? (knowledge and perceptions on energy, role in mitigating the effects of climate change through efficient use of energy)

What experiences/issues do you face concerning promoting efficient energy use in people`s homes? Probe: Knowledge, attitudes, locus of control,

What are some of the positive and negative experiences concerning energy use people`s homes homes? Explain what caused these

What challenges do you face concerning energy use in your homes?

What are your fears concerning energy use in your neighbourhood?

What is the role of the household head(s), children, elders concerning how you use energy in the homes? What have they done to control how you use energy in your homes?

(Would you now consider yourself to have a responsibility on how energy is used in your homes? If not, what would you do to save energy if you had the responsibility?)

What do you think are the roles of government, agencies, officials, local leadership concerning energy and energy use in your homes and locality?

What laws or regulations do you know concerning energy and energy use in your locality?

In your own opinion, what are some of the implications of global changes (things happening in other parts of the world) on efficient use of energy by children and young people? Probe:

Globalization (interconnectedness and interdependentness of the world), technological innovations and increasing population, migration, urbanization etc.

Can you share your suggestions and recommendations on how authorities can make it easier for children and young people to use energy efficiently?

Preferred learning methods vs. contemporary methods

Policy and governance

2. SBC communication platforms

Where/How do you get information on Efficient energy use in your community?

Probe:—Radio, TV, newspapers, local leadership, authorities (EMA), community leaders, school teachers, other young people)

What promotional strategies, activities, messages, and message channels are most likely to increase adoption of behaviours related to efficient use of energy?

How best can organisations disseminate information on Efficient use of energy in your community?

3. Landscape mapping

Please explain organisations that are working to promote efficient use of energy in this community and their role—Probe -name of organisation, area of operation, activities by the organisation, effectiveness

4. SBC skills gap analysis (for government workers, NGO/CSO staff)

Please explain the skills that you have concerning Social and behavioural issues around promoting open defecation free communities/proper and consistent use of latrines. Probe formal and informal training

What Social and behavioural change skills would make your job easier/will result in increase in proper and consistent use of latrines?
